# Exploring molecular markers and drug candidates for colorectal cancer through comprehensive bioinformatics analysis

**DOI:** 10.18632/aging.204891

**Published:** 2023-07-18

**Authors:** Guangyao Li, JiangPeng Zhu, Lulu Zhai

**Affiliations:** 1 Department of Gastrointestinal Surgery, The Second People’s Hospital of Wuhu, Wuhu, Anhui, People’s Republic of China; 2 Department of General Surgery, Renmin Hospital of Wuhan University, Wuhan 430060, Hubei, People’s Republic of China

**Keywords:** colorectal cancer, WGCNA, biomarker, prognosis, drugs

## Abstract

Colorectal cancer (CRC) often has a poor prognosis and identifying useful and novel agents for treating CRC is urgently required. This study aimed to examine molecular markers associated with CRC prognosis and to identify potential drug candidates. The differentially expressed genes (DEGs) of CRC in TCGA were identified. The genes associated with CRC, summarized from NCBI-gene, OMIM, and the DEGs, were used to construct a co-expression network by WGCNA. Moreover, the co-expression genes from modules of interest were used to carry out functional enrichment. A total of 2742 DEGs, including 1674 upregulated and 1068 downregulated genes, were identified. Thirteen co-expression modules were constructed with WGCNA. Brown and blue co-expression modules with significant differences in disease phenotype were found. Functional enrichment analysis showed that genes in the brown module were mainly related to cell cycle, cell proliferation, DNA replication, and RNA transport. The genes in the blue module were mainly associated with fatty acid degradation, sulfur metabolism, PPAR signaling pathway and bile secretion. In addition, both the genes in brown and blue were associated with tumor staging. Some prognostic markers and candidate small molecules drugs for CRC treatment were identified. In conclusion, we revealed molecular biomarker profiles in CRC by systematic bioinformatics analysis, constructed regulatory networks of mRNA, ncRNA and transcriptional regulators (TFs), and identified potential drugs targeting hub proteins and TFs.

## INTRODUCTION

Colorectal cancer (CRC) is one of the leading malignancies in humans, ranking in the top three in terms of incidence and mortality, accounting for approximately 10% of all cancer cases and deaths [1, 2]. By 2030, the new cases and deaths are forecasted to more than 2.2 million and 1.1 million, respectively [3]. The introduction of new chemotherapeutics, along with improving techniques in biological treatment, have significantly improved survival rates. However, elucidating the mechanisms of tumorigenesis and progression and searching for effective drugs in CRC remains challenging. Thus, it is important to identify novel therapeutic target molecules and drugs.

Bioinformatics has emerged as a potential tool to understand the mechanisms of gene regulation and identify agents [4–6]. Weighted gene co-expression network analysis (WGCNA), a systematic biological method to identify the relationships between genes and phenotypes, allows the exploration of modules that are candidate regulators and drivers of disease states [7, 8]. The objective of this work was to examine the key genes involved in tumorigenesis and progression in CRC through WGNCA and to identify potential therapeutic drugs.

## MATERIALS AND METHODS

### Extraction and processing of data

RNA sequencing data and clinic traits of patients were obtained from The Cancer Genome Atlas (TCGA) database (https://cancergenome.nih.gov/). A total of 464 CRC samples and 41 normal samples were included. The gene probe IDs were matched to the gene symbols using Perl language command. Before Log2 transforming, R package was used to transform FPKM values into TPM values. Moreover, Human CRC genes were downloaded from the NCBI (https://www.ncbi.nlm.nih.gov/) as well as OMIM site (https://www.omim.org/). All cancer-related medications were collected from the DrugBank database (https://go.drugbank.com/). Single-Cell data were downloaded from CancerSEA (http://biocc.hrbmu.edu.cn/CancerSEA).

### WGCNA analysis

Two obvious outliers were removed from the cohort. Then, the weighted gene co-expression network was constructed with WGCNA package in R software [9, 10]. The gene expression profiles were constructed using the genes associated with CRC summarized from NCBI-gene, OMIM, and the differentially expressed genes (DEGs) of TCGA. The WGCNA algorithm was carried out to identify different modules. The correlation between the different modules and phenotype was then analyzed with Pearson correlation coefficient [11].

### Functional and pathway enrichment analyses of co-expression modules

The functional and pathway enrichment analyses of genes in the constructed modules were performed employing Cluster profiler R package (*P* value cutoff = 0.01, *q* value cutoff = 0.01).

### PPI network construction

The PPI (protein-protein interaction) information for hub modules was explored via STRING (Search Tool for the Retrieval of Interacting Genes Database) (http://www.string-db.org/) [12].

### Network module cross-talk analysis

Interactions between genes play an important role in certain biological functions. As such, identification of the interactions between modules is important for understanding how these genes interact with other genes and sub-networks. To elucidate the interactions between modules, the human PPI information was used as a background set and a comprehensive cross-talk analysis was implemented for all modules to further understand the interaction mechanism of co-expression modules. First, the human protein interaction network (score > 900) in String was used to generate 1000 random networks while keeping the network size and the degree of each node unchanged. Then, the number of interaction pairs between modules was counted according to the random network. Finally, the number of interaction pairs between modules is compared with the number of interaction pairs in a random background. When the number of interaction pairs between modules is greater than the number of interaction pairs in a random background, these interactions are called cross-talk. In the context of random networks, if the number of interaction pairs between modules in N random networks is greater than the number of interaction pairs between modules in the real network, then the number is recorded as n. The formula for calculating *p* value: *p* = n/N (*N* = 1000). When the *p* value < 0.05, it can be considered that these cross-talk modules are more significant than random ones. In other words, there is a cross talk interaction between modules [13, 14].

### Pivot analysis predicts module transcriptional regulators and potential drugs

The pivot node is defined as follows: (i) There are at least two pairs of interactions with the module gene; (ii) The *p* value of the significance analysis of the interaction between the node and each module should be ≤ 0.05, and the statistical method is hypergeometric test. The pivot node of the interaction module for further analysis was explored with Python program. In general, Gene transcription and post-transcriptional regulation are driven by non-coding RNA (ncRNA) and transcriptional regulators (TFs). Hence, ncRNA and TFs were predicted and their roles were detected in CRC-related dysfunction modules.

The analysis method of ncRNA pivot: The interaction relationship of ncRNA-mRNA included in the RAID 2.0 database was used as the interaction background, all the interaction pairs between ncRNA and modular genes are counted. Then the interaction pairs between each ncRNA and the genes in or outside the module were counted. Pivot was selected according to the significance *p*-value of the hypergeometric test. Using the same way, Pivots of TFs and drugs in the TRRUST v2 database and DrugBank database were selected, respectively [14]. The complete analysis flow was presented in Supplementary Figure 1.

### Availability of data and materials

The datasets used and/or analyzed during the current study are available from the corresponding author on reasonable request.

## RESULTS

### DEGs screening of CRC

Gene expression profiles of 464 tumors and 41 corresponding normal tissues were obtained from TCGA. Using |log2FC| > 1 and FDR < 0.05 as the threshold, 2742 DEGs, including 1674 upregulated and 1068 downregulated genes, were screened out.

### Weighted co-expression network construction and key modules identification

**Figure 1:**
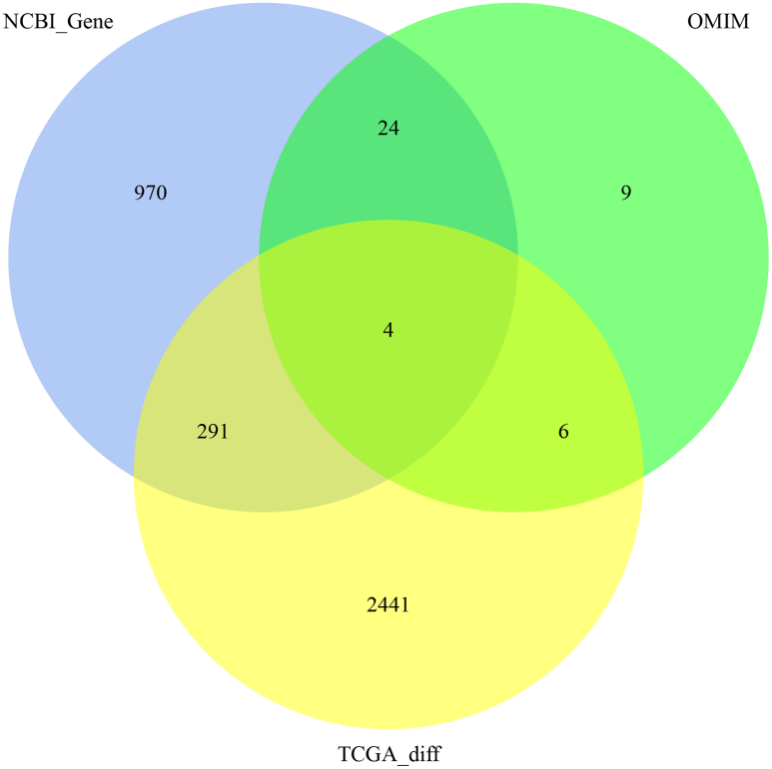
3745 genes associated with CRC in NCBI-gene, OMIM, and the DEGs of TCGA.

**Figure 2:**
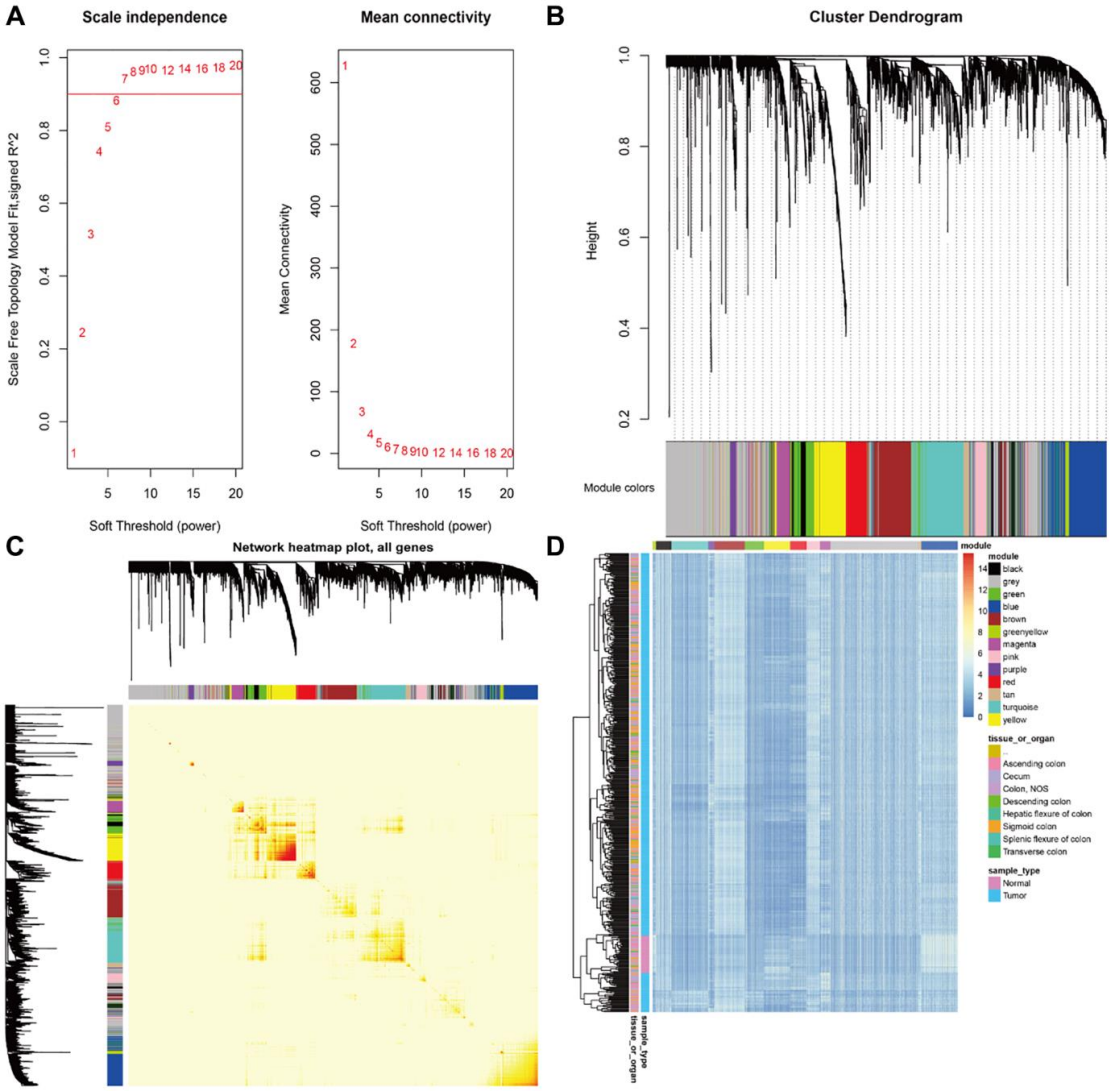
(**A**) The network parameter selection. (**B**) The cluster dendrogram of the differentially expressed genes. (**C**) Identification of modules associated with the clinical traits. Interaction relationship analyses of co-expression genes. Different colors of horizontal axis and vertical axis represent different modules. The brightness of yellow in the middle represents the degree of connectivity of different modules. There was no significant difference in interactions among different modules, indicating a high-scale independence degree among these modules. (**D**) The samples were mainly divided into two clusters according to the gene expression in the gene modules.

**Figure 3:**
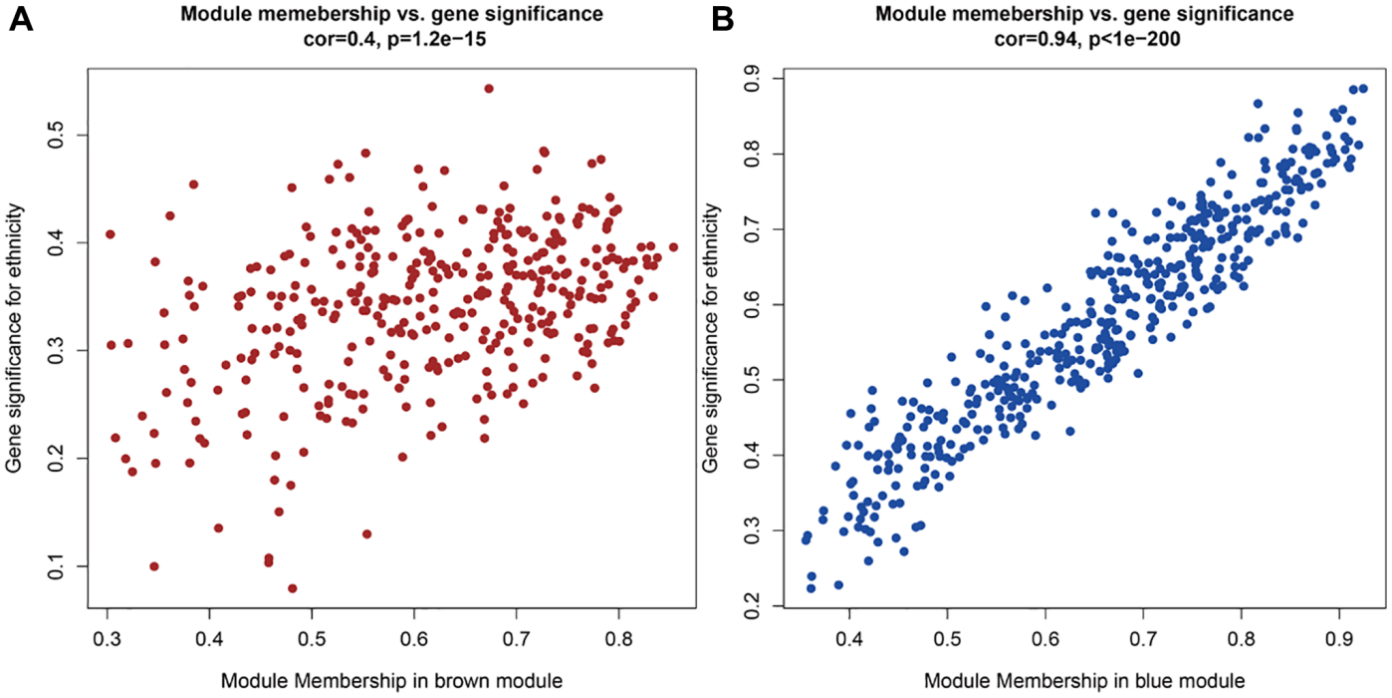
**The genes in this two module correlations with the phenotype of CRC.** (**A**) The genes in the brown module correlations with the phenotype of CRC. (**B**) The genes in the blue module correlations with the phenotype of CRC.

A total of 3745 genes associated with CRC were used to construct a co-expression network by WGCNA (Figure 1). And of them, 1289 genes were downloaded from the NCBI-gene database, 43 genes were downloaded from the OMIM database and 2742 DEGs were screened out from TCGA. When co-expression analysis was performed by WGCNA, clustering showed that 2 samples were outliers. After removing these 2 samples, the remaining 503 samples were analyzed. We set the power β to a soft-thresholding parameter β = 8 (scale free R^2^ = 0.9) to ensure a scale-free network (Figure 2A). Then we selected a soft threshold of 7 in the network construction process and 13 modules were identified (Figure 2B, 2C).The samples were clustered according to the gene expression in the gene modules and all tumor samples were mainly divided into two clusters (Figure 2D). The CRC phenotypes (Normal and Tumor) in the two types of sample modules were counted separately, and the chi-square test was performed. The results showed that the two types of samples have significant differences in the CRC phenotype (Table 1, *P* value = 2.2e-16), suggesting these modules were related to the tumorigenesis of CRC. In order to determine the correlation between gene modules and CRC phenotype, the characteristic value of each gene module were calculated. Then, the correlation with the sample phenotype (Normal and Tumor) and the *P* value of the corresponding correlation were calculated. The results of gene module characteristic values and phenotypic correlation coefficients are shown in Table 2. Among them, the most positively correlated module is brown, and the most negatively correlated module is blue. Moreover, the brown and blue modules were significantly associated with CRC phenotype compared with other modules (Figure 3).

**Table 1:** The distribution of tumor samples in cluster 1 and cluster 2.

	**Normal**	**Tumor**
Cluster 1	0	418
Cluster 2	41	45

**Table 2:** Gene module characteristics and phenotype correlation results.

**Module**	**Cor**	***P*-value**
Black	0.524539269	5.66E-37
Purple	0.270138429	7.07E-10
Brown	0.538135736	3.52E-39
Pink	0.479827937	2.20E-30
Tan	0.388179852	1.43E-19
Blue	−0.883369869	2.42E-167
Green yellow	−0.585090335	1.27E-47
Green	−0.185033296	2.92E-05
Turquoise	0.272366704	5.06E-10
Magenta	0.202002654	4.86E-06
Red	−0.380699969	7.91E-19
Yellow	−0.5373022	4.84E-39
Grey	0.570203511	8.46E-45

### Modular genes detection and validation

**Figure 4:**
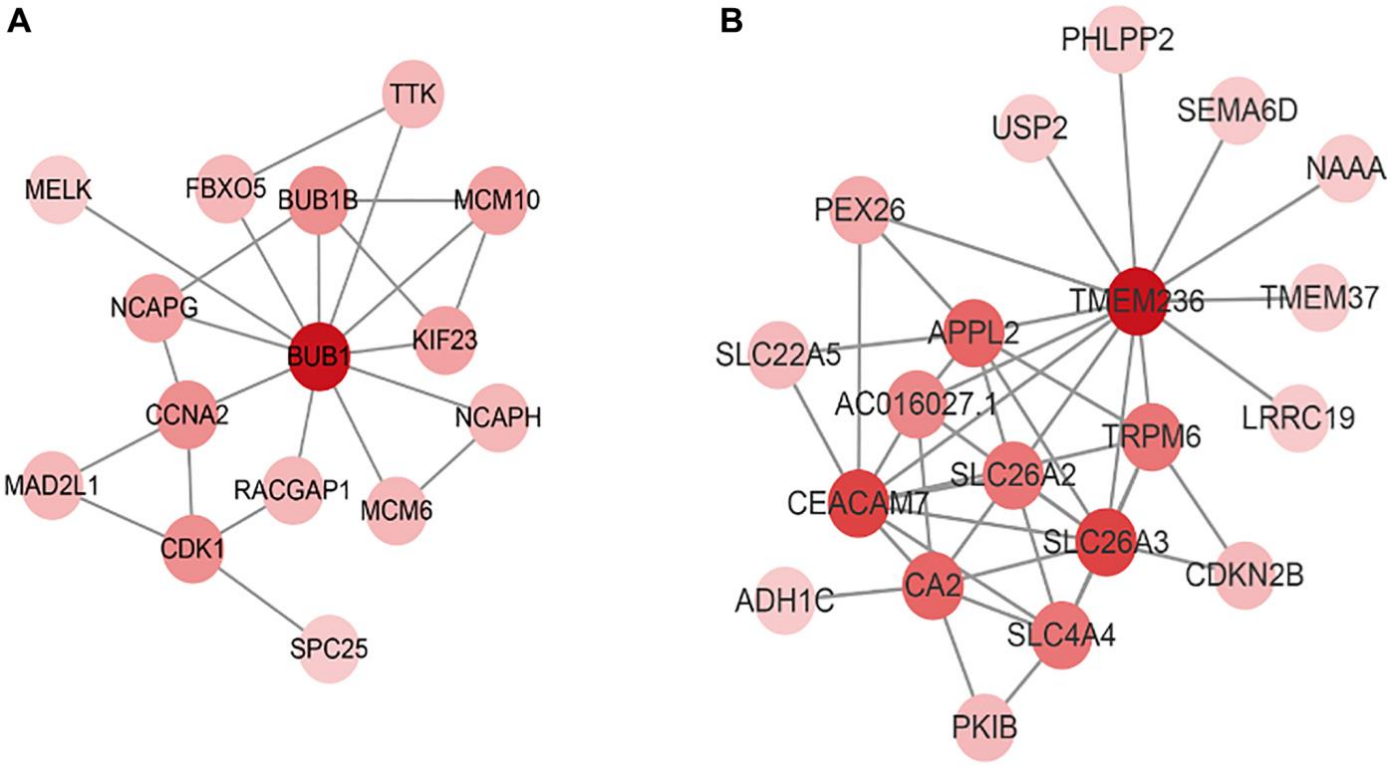
**The network of candidate tumor driver genes in two modules.** (**A**) The network of candidate tumor driver genes in brown module. (**B**) The network of candidate tumor driver genes in blue module.

**Figure 5:**
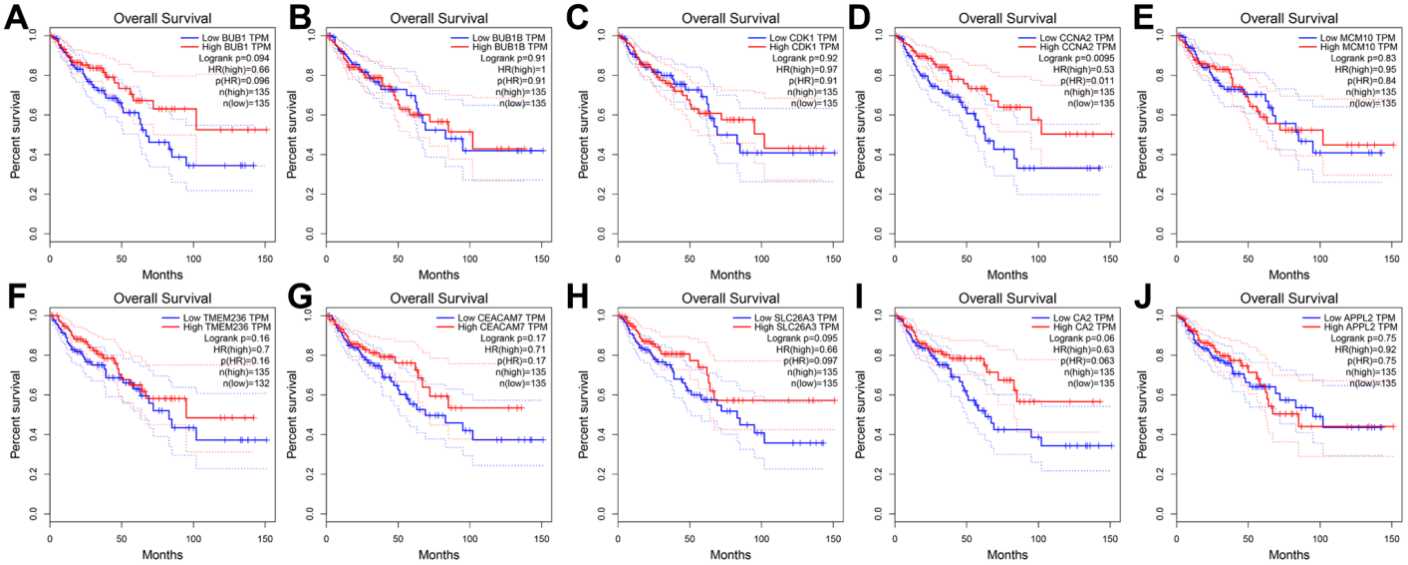
**Overall survival analysis of 10 key genes in CRC (based on TCGA data in GEPIA).** (**A**–**J**) Expression levels of CCNA2 are significantly related to the overall survival of patients with CRC (*P* < 0.05).

**Figure 6:**
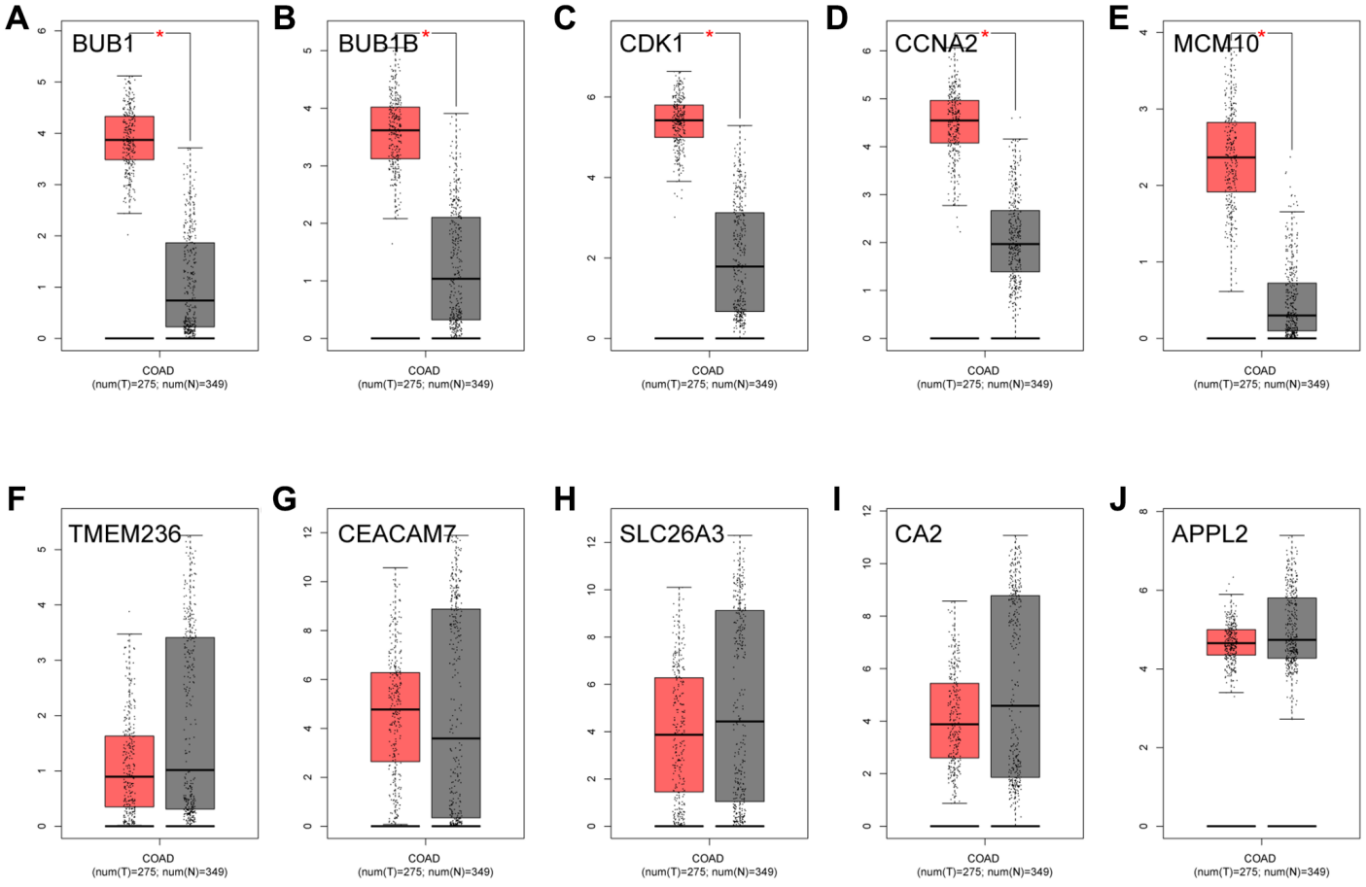
**The expression analysis of 10 key genes in CRC (based on TCGA data in GEPIA).** (**A**) BUB1, (**B**) BUB1B, (**C**) CDK1, (**D**) CCNA2, (**E**) MCM10, (**F**) TMEM236, (**G**) CEACAM7, (**H**) SLC26A3, (**I**) CA2, (**J**) APPL2.

**Figure 7:**
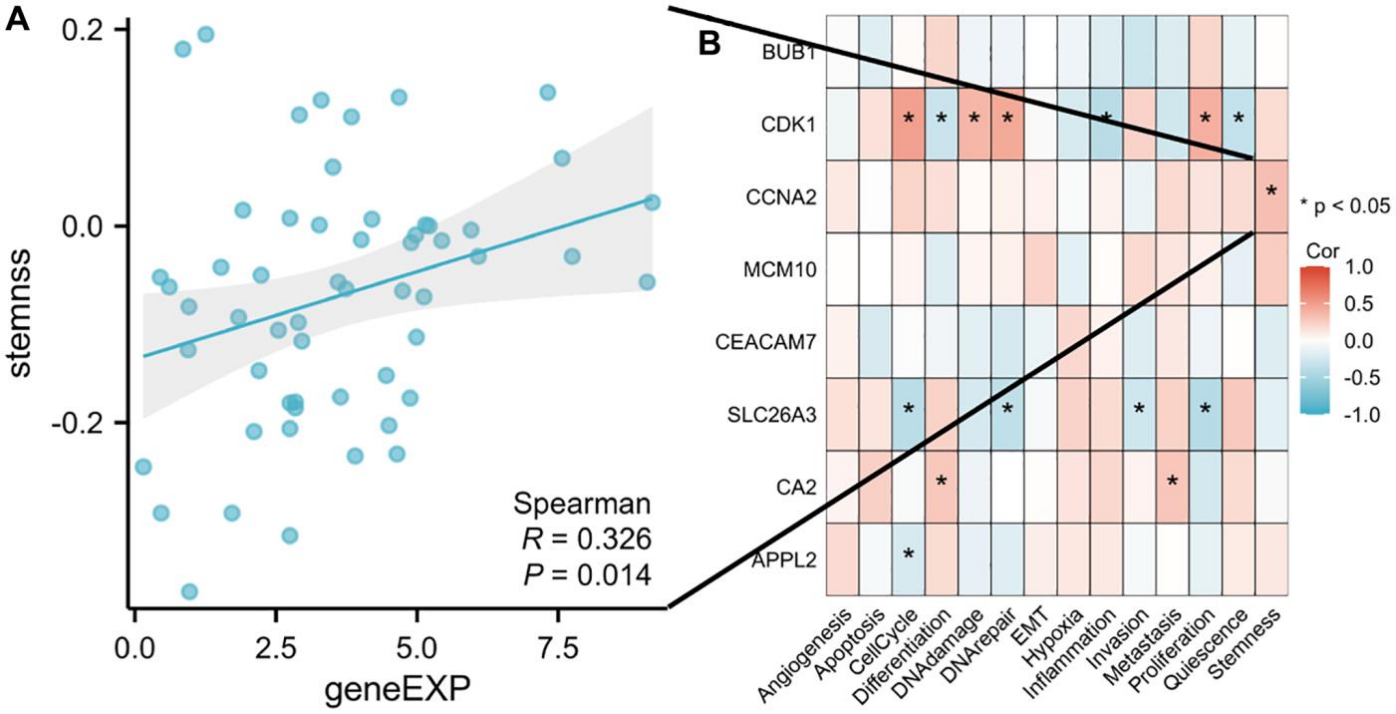
**The function of hub genes in single-cell functional analysis from the CancerSEA database.** (**A**) Scatter plot of the correlation between CCNA2 and stemness in CRC. (**B**) Heat map of correlation between hub genes and functional status in CRC. (^*^p < 0.05).

To identify the functions of brown and blue modules, the core genes of the two modules need to be further analyzed to determine the core driver genes of tumor deterioration. We visually displayed the genes in the two modules (Figure 4), calculated the degree of each gene node, and selected genes with high screening degrees as candidate tumor driver genes. According to the degree of connectivity, the top five genes in brown module were BUB1, BUB1B, CDK1, CCNA2, MCM10 and the top five genes in blue module were TMEM236, CEACAM7, SLC26A3, CA2, and APPL2. The above 10 modular genes were then validated with CRC data of GEPIA database. Among them, CCNA2 was negatively associated with the overall survival of CRC patients (Figure 5). Moreover, the expression levels of BUB1, BUB1B, CDK1, CCNA2, MCM10 were significantly higher in CRC tumor tissues compared with normal tissues based on the GEPIA database (Figure 6). The roles of BUB1B, CDK1, CCNA2, MCM10, CEACAM7, SLC26A3, CA2, and APPL2 in CRC development were explored based on scRNA-seq data of CancerSEA database. The results showed that these hub genes were positively related to cell cycle, DNA damage, DNA repair, proliferation, stemness, differentiation, and metastasis. For example, CCNA2 was positively related to stemness in CRC (Figure 7).

### Functional annotation and KEGG pathway enrichment of key modules

**Figure 8:**
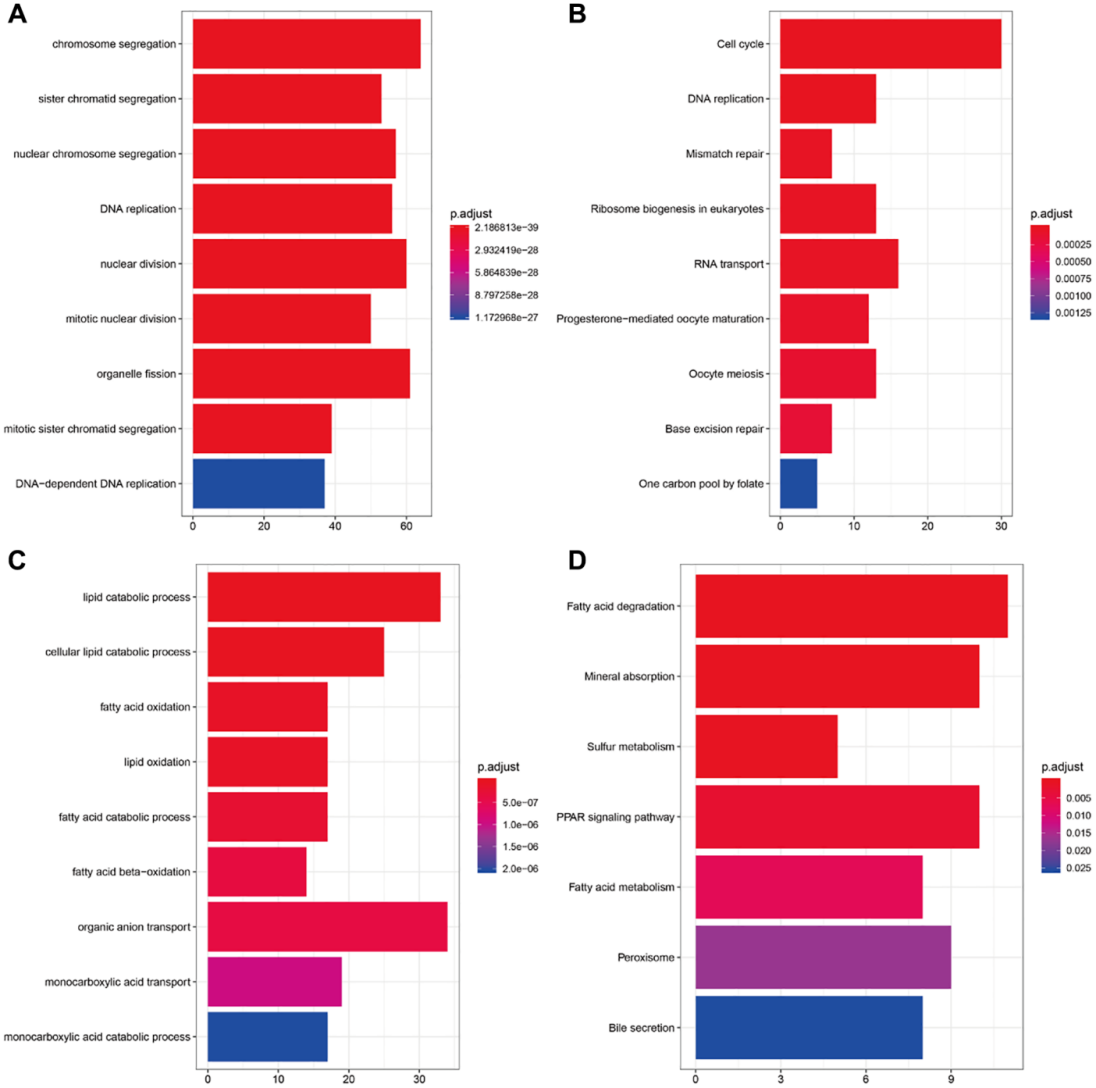
**Enrichment analysis of brown and blue modules.** (**A**) GO analysis of all genes in brown modules. (**B**) KEGG pathway analysis of all genes in brown modules. (**C**) GO analysis of all genes in blue modules. (**D**) KEGG pathway analysis of all genes in blue modules.

Gene ontology (GO) and Kyoto Encyclopedia of Genes and Genomes (KEGG) pathway enrichment analyses of the brown and blue modules were performed to explore potential biological processes and mechanisms associated with CRC. The result showed that the genes in the brown modules were mainly related to chromosome segregation, nuclear division, organelle fission and other functions in go enrichment (Figure 8A), while related to cell cycle, DNA replication and RNA transport and other pathways in KEGG pathway enrichment. (Figure 8B). Meanwhile, the genes in the blue modules were mainly related to lipid catabolic process, cellular lipid catabolic process, organic anion transport and other functions in go enrichment (Figure 8C), while related to fatty acid degradation, mineral absorption, PPAR signaling pathway, and other pathways in KEGG pathway enrichment (Figure 8D).

### Module cross-talk analysis

**Figure 9:**
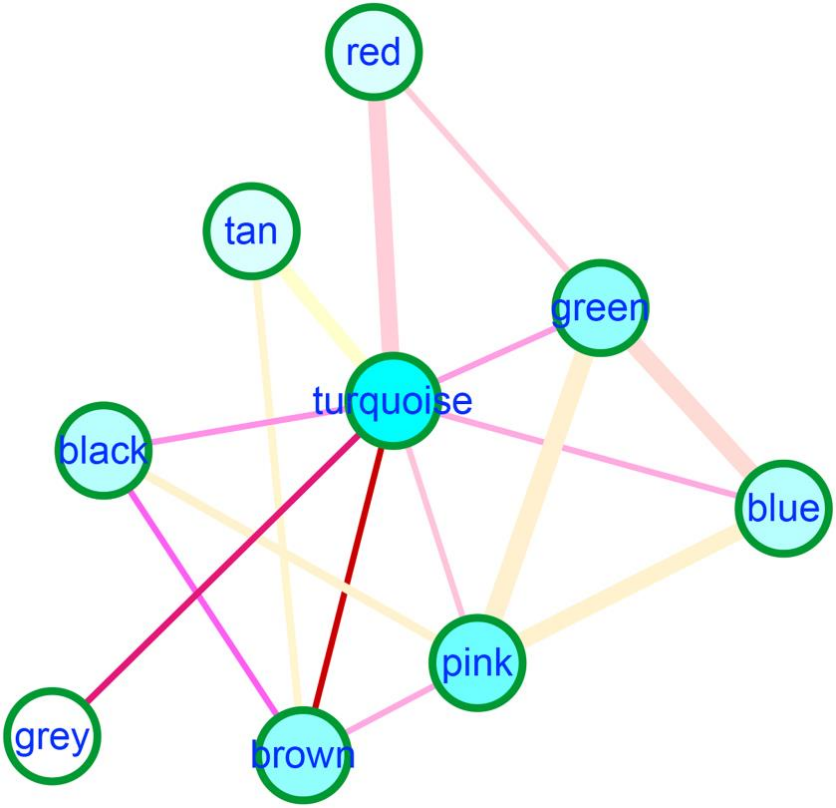
The module crosstalk analysis showed blue and brown modules had strong interactions with other modules.

With the human protein interaction network as the background, we constructed 1000 random networks and calculated the interaction pairs between each gene module. We set the significance threshold *P* value < 0.05 and screened 16 significant cross talks (module cross-talk interaction pairs). The blue and brown modules had strong interactions with other modules (Figure 9).

### ncRNA regulating modular genes

**Figure 10:**
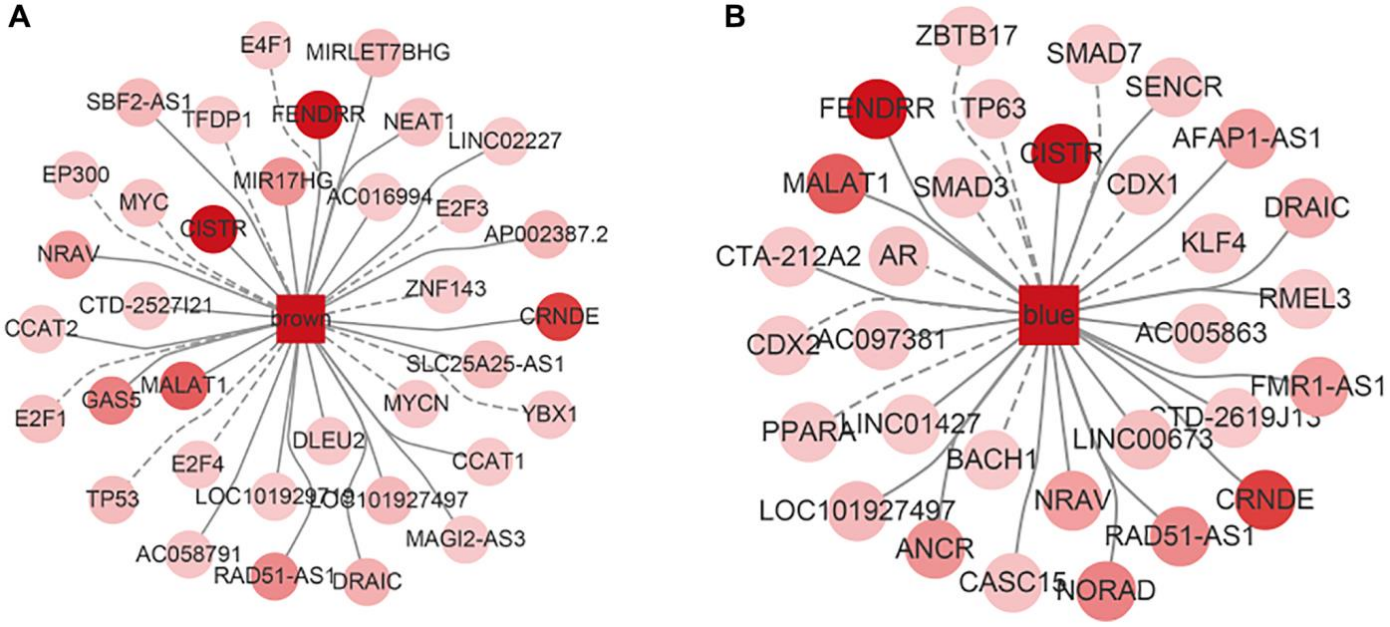
**The interaction network among this two module, lncRNA and TF.** The darker the color, the more significant the interaction with the module, the dashed line represents TF, the solid line represents ncRNA. (**A**) The network in brown module. (**B**) The network in blue module.

Based on the 70,962 pairs of ncRNA-mRNA interaction relationship included in the RAID 2.0 database, we explored the pivot node (ncRNA) of the regulatory function modules. When *P* value < 0.01, a total of 260 Pivot-Module interaction pairs were screened out, including 164 ncRNAs. The results were shown in Table 3 (only the modules significantly related to the phenotype were listed), with each module displaying the most significant 5 Pivot-Module interaction pairs. The ncRNAs that had significant regulatory effects on the brown module included MALAT1, CRNDE, FENDRR, DRAIC, LOC101927497, etc. The ncRNAs with significant regulatory effects on the blue module included ANCR, AFAP1-AS1, CISTR, FMR1-AS1, MALAT1, etc., (Figure 10).

**Table 3:** Pivot (ncRNA)-module interaction pairs.

**Module**	**ncRNA**	**Connection**	***P*-value**
Brown	MALAT1	1569	2.88E-23
Brown	CRNDE	1984	2.36E-21
Brown	FENDRR	2672	8.23E-16
Brown	DRAIC	386	5.94E-12
Brown	LOC101927497	237	1.23E-11
Blue	ANCR	795	1.23E-20
Blue	AFAP1-AS1	590	9.38E-15
Blue	CISTR	2883	2.32E-14
Blue	FMR1-AS1	619	1.41E-10
Blue	MALAT1	1569	5.15E-10
Black	CISTR	2883	1.45E-08
Black	FENDRR	2672	1.68E-08
Black	MALAT1	1569	3.54E-08
Black	MIR17HG	800	4.10E-07
Black	NRAV	622	7.90E-06
Green	FENDRR	2672	3.61E-12
Green	AFAP1-AS1	590	3.35E-09
Green	MALAT1	1569	6.12E-09
Green	CRNDE	1984	9.24E-06
Green	H19	29	3.73E-05
Green yellow	C8orf34-AS1	46	4.00E-04
Green yellow	AC079779	2	1.27E-03
Green yellow	RP11-834C11	2	1.27E-03
Green yellow	SNHG3	2	1.27E-03
Green yellow	LINC01852	3	1.90E-03
Grey	ANCR	795	2.23E-27
Grey	AFAP1-AS1	590	3.67E-20
Grey	CISTR	2883	1.42E-18
Grey	FENDRR	2672	6.20E-11
Grey	H19	29	5.88E-09
Magenta	AFAP1-AS1	590	2.54E-08
Magenta	HOXA11-AS	8	3.17E-08
Magenta	PANDAR	3	9.97E-08
Magenta	ANCR	795	2.73E-07
Magenta	CISTR	2883	8.29E-07
Pink	CISTR	2883	1.31E-09
Pink	SNHG16	723	1.41E-05
Pink	RAD51-AS1	905	1.20E-04
Pink	MIR17HG	800	1.69E-04
Pink	CRNDE	1984	3.28E-04
Purple	NRAV	622	2.59E-06
Purple	CISTR	2883	2.02E-05
Purple	LOC284191	2	1.94E-03
Purple	CALML3-AS1	3	2.91E-03
Purple	AP000265	4	3.88E-03
Red	CASC15	120	1.21E-07
Red	CAT8	190	2.68E-06
Red	NORAD	1028	4.61E-04
Red	AFAP1-AS1	590	6.12E-04
Red	GAS5	1067	6.25E-04
Tan	CISTR	2883	1.93E-04
Tan	FENDRR	2672	1.83E-03
Tan	LA16c-380H5	2	2.08E-03
Tan	LOC102724908	2	2.08E-03
Tan	RP1-101D8	2	2.08E-03
Turquoise	KCNJ2	7	5.14E-04
Turquoise	DEPDC1	5	1.10E-03
Turquoise	MAPRE1	5	1.10E-03
Turquoise	ATG101	3	1.95E-03
Turquoise	CYB561D2	3	1.95E-03
Yellow	FENDRR	2672	8.10E-20
Yellow	CRNDE	1984	1.57E-17
Yellow	ANCR	795	2.20E-13
Yellow	LOC101927497	237	5.26E-09
Yellow	MALAT1	1569	1.86E-06

### TFs regulating modular genes

Based on the 9396 pairs of TFs-mRNA interaction relationship included in the TRRUST v2 database, we explored the pivot node (TFs) of the regulatory function modules. When *P* value < 0.01, a total of 80 Pivot-Module interaction pairs were screened out, including 77 TFs. The results were shown in Table 4 (only the modules significantly associated with the phenotype were listed), with each module displaying the most significant 5 Pivot-Module interaction pairs. The TFs that had significant regulatory effects on the brown module included E2F1, MYC, TP53, E2F4, YBX1, etc. The TFs with significant regulatory effects on the blue module included ZBTB17, CDX2, PPARA, AR, CDX1, etc., (Figure 10).

**Table 4:** Pivot (TFs)-module interaction pairs.

**Module**	**TFs**	**Connection**	***P*-value**
Brown	E2F1	155	1.96E-15
Brown	MYC	113	4.13E-08
Brown	TP53	201	1.54E-07
Brown	E2F4	26	3.79E-05
Brown	YBX1	33	1.96E-04
Blue	ZBTB17	3	1.19E-04
Blue	CDX2	36	2.92E-04
Blue	PPARA	43	4.65E-03
Blue	AR	105	5.38E-03
Blue	CDX1	8	5.53E-03
Black	FLI1	26	7.22E-03
Black	HDAC5	10	9.81E-03
Black	LMO2	10	9.81E-03
Green	STAT3	185	4.17E-03
Green	NFKBIA	23	6.18E-03
Green	WWP1	3	8.08E-03
Green	ZFP36	9	9.72E-03
Green yellow	NR1I2	27	1.93E-05
Green yellow	EAPP	4	9.39E-04
Green yellow	NR1I3	5	1.55E-03
Green yellow	NR0B2	9	5.40E-03
Green yellow	YBX1	33	8.19E-03
Grey	ATF4	41	7.29E-04
Grey	SMARCA4	9	2.30E-03
Grey	ARNT	22	4.12E-03
Grey	DNMT1	33	5.12E-03
Grey	ZNF24	5	5.50E-03
Magenta	KLF8	8	1.50E-05
Magenta	RUNX2	21	4.28E-04
Magenta	SMAD3	35	1.47E-03
Magenta	TWIST2	27	1.81E-03
Magenta	CITED2	2	2.49E-03
Pink	RB1	35	2.22E-04
Pink	ENO1	5	6.12E-03
Pink	MED1	5	6.12E-03
Pink	PA2G4	5	6.12E-03
Pink	ARID3A	6	9.03E-03
Purple	HIF1A	95	6.25E-03
Purple	PIAS1	6	7.64E-03
Red	NFKB1	399	4.96E-06
Red	RELA	394	4.04E-05
Red	STAT1	98	3.40E-04
Red	IRF1	57	1.12E-03
Red	NFKBIA	23	1.35E-03
Tan	RBL1	5	4.78E-03
Turquoise	KAT2B	13	1.90E-03
Turquoise	TP53BP1	2	1.92E-03
Turquoise	RB1CC1	3	5.59E-03
Turquoise	JUND	38	5.74E-03
Yellow	ARNTL	10	1.65E-06
Yellow	CLOCK	18	4.79E-05
Yellow	NPAS2	4	3.26E-03
Yellow	EZH2	43	3.32E-03
Yellow	HOXD3	5	5.35E-03

### Drug candidates for module genes

We retrieved 17 drugs to treat CRC from the DrugBank database, and we searched for the drug pivot nodes of the regulatory function modules using drug-gene interactions as a background set. When *P* < 0.05, a total of 3 Pivot-Module interaction pairs were obtained, including 3 drugs. We did not yet detected drugs that were significantly related to the brown and blue modules (Table 5). Nevertheless, it was clear from the above analysis that the genes in the brown and blue modules may play an important role in the phenotype of CRC. To explore more effective drugs for the treatment of CRC, we screened the drugs of the brown and blue regulatory modules in the context of all drug-gene interaction pairs in the DrugBank. The results were shown in Table 6 (top 5, sorted by regulatory interaction pairs).

**Table 5:** Pivot (drugs)-module interaction pairs.

**Module**	**Pivot**	***n***	***P*-value**
Grey	Mepenzolate	2	0.008032
Red	Olsalazine	2	0.02381
Yellow	Trimebutine	21	0.012873

**Table 6:** The drugs related to modules brown and blue.

**Module**	**TFs**	**Connection**	***P*-value**
Blue	L-Carnitine	14	3.43E-06
Blue	Oleic-Acid	12	2.80E-05
Blue	Creatine	7	1.21E-03
Blue	2-[(2,4-DICHLOROBENZOYL) AMINO]-5-(PYRIMIDIN-2-YLOXY) BENZOIC-ACID	3	3.33E-03
Blue	2-chloro-5-nitro-N-phenylbenzamide	3	3.33E-03
Brown	1-(3,5-DICHLOROPHENYL)-5-METHYL-1H-1,2,4-TRIAZOLE-3-CARBOXYLIC-ACID	2	5.45E-04
Brown	1-[4-(AMINOSULFONYL) PHENYL]-1,6-DIHYDROPYRAZOLO [3,4-E] INDAZOLE-3-CARBOXAMIDE	2	5.45E-04
Brown	1-methyl-8-(phenylamino)-4,5-dihydro-1H-pyrazolo [4,3-h] quinazoline-3-carboxylic-acid	2	5.45E-04
Brown	2-ANILINO-6-CYCLOHEXYLMETHOXYPURINE	2	5.45E-04
Brown	(2R)-2-{[4-(benzylamino)-8-(1-methylethyl) pyrazolo [1,5-a] [1,3,5] triazin-2-yl] amino} butan-1-ol	2	5.45E-04

## DISCUSSION

CRC is the most common gastrointestinal tumor and its progression is related to the activation of various oncogenes and the inactivation of various tumor suppressor genes [15, 16]. With the rapid development of bioinformatics, databases are widely used to analyze tumor-related molecules and explore therapeutic agents [17, 18]. In this study, we obtained biomarker profiles of CRC through comprehensive bioinformatics analysis, investigated the biological processes and mechanisms involved in these biomarkers, constructed regulatory networks of mRNA, ncRNA and TFs, and identified potential drug candidates targeting hub proteins and TFs.

In the present study, WGCNA analysis showed that the brown and blue modules were significantly associated with CRC phenotype compared with other modules. GO enrichment analysis showed that the genes in the brown module were significantly enriched in cell cycle, DNA replication, RNA transfer, etc. DNA replication has been thought as a source for gene amplification in tumors. Available studies confirmed that the above mentioned entries were related to tumor development [19, 20]. Meanwhile, GO analysis suggested that the genes in the blue module were mainly enriched in fatty acid degradation, sulfur metabolism, PPAR signaling pathway, etc. The reduction in fatty acids often leads to the inhibition of tumor cells proliferation [21]. However, whether the PPAR signaling pathway acts as a pro- or anti-tumor agent in CRC is currently controversial and needs to be explored in depth [22].

We then determined the core driver genes of tumor deterioration in the brown and blue modules. The top five genes were BUB1, BUB1B, CDK1, CCNA2 and MCM10 in the brown module and TMEM236, CEACAM7, SLC26A3, CA2 and APPL2 in the blue module. Among the core genes, BUB1 was identified as a risk factor for CRC [23]. CDK1 was shown to regulate CRC cell proliferation through p53 pathway [24]. CCA2 was reported to be a prognostic factor for CRC [25]. SLC26A3 has been shown to be an important tumor suppressor gene in CRC [26]. In addition, elevated CA2 suppressed tumor cell growth both *in vitro* and *in vivo* [27]. However, the roles of BUB1B, MCM10, TMEM236, CEACAM7 and APPL2 in CRC have not been reported and further investigated are needed.

We also explored the potential ncRNAs based on the ncRNA-mRNA interaction relationships included in the RAID 2.0 database. There are some ncRNAs that have been shown to be associated with tumor progression. For example, aberrant expression of MALAT1 was involved in tumor angiogenesis and metastasis [28, 29]. CRNDE regulated the invasion and migration of CRC through the Wnt/β-catenin signaling pathway [30]. FENDRR influenced CRC progression via regulating miR-18a-5p and miR-424-5p [31, 32]. ANCR modulated CRC progression by binding specifically to EZH2 [33]. AFAP1-AS1 affected CRC progression through miR-195-5p/WISP1 axis [34]. In addition, DRAIC, LOC101927497, CISTR and FMR1-AS1 were thought to be closely related to the progression of other tumors [35, 36]. However, their role in CRC remains to be explored.

Moreover, we obtained TFs of the regulatory function modules based on the TRRUST v2 database. Some TFs are associated with tumor progression, including CRC. For instance, E2F1 regulated CRC progression and contributed to oxaliplatin resistance [37, 38]. MYC influenced the cell cycle and tumorigenesis by regulating lncRNAs [39]. CRC with stabilized mutp53 exhibited enhanced Jak2/Stat3 signaling and were associated with poorer survival [40]. E2F4 was deemed to be an important target gene in the regulation of CRC carcinogenesis [41]. PRKCQ-AS1 impacted CRC progression by regulating miR-1287-5p/YBX1 pathway [42]. A targeted proteomics approach has revealed ZBTB17 serves as a diagnostic marker for resectable gastric cancer [43]. CDX2 was involved in the epithelial-mesenchymal transition of CRC through PTEN [44]. The functionally related FOXM1 and PPARA were implicated in the vascular endothelial growth factor receptor signaling pathway in CRC [45]. CDX1 affected CRC differentiation and was regulated by promoter methylation [46]. However, the relationship between AR and tumors, particularly between it and CRC, needs to be further investigated.

Finally, we found that L-carnitine and oleic-acid could serve as potential therapeutic agents for CRC. However, L-carnitine mediated cytoprotection in glioblastoma multiforme and led to poor prognosis of patients [47]. Moreover, L-carnitine was involved in the pathogenesis of endometrial cancer [48]. Studies are lacking to explain whether there is a relationship between L-carnitine and the development of CRC. Oleic-acid promoted CRC metastasis following the induction of NOX4 [49].

The innovation of this work compared to former studies is the discovery of several new molecules and drug candidates not reported in CRC through the use of a comprehensive set of genes. However, there are still several limitations. First, the data in this study were only obtained from public databases and further validation of clinical specimens is needed. Second, we did not explore the impact of the identified molecular markers on CRC progression. Finally, we did not investigate the anti-tumor effects of the identified drug candidates *in vivo* and *in vitro*. Therefore, future studies need to collect more clinical specimens and design more experiments to further functional validation of the identified molecular markers and drug candidates.

## CONCLUSION

In conclusion, we revealed molecular biomarker signatures at the RNA and protein levels in CRC by systematic bioinformatics analysis, and constructed regulatory networks for mRNAs, ncRNAs and TFs. Moreover, we identified potential drugs targeting hub proteins and TFs.

## Supplementary Materials

Supplementary Figure 1
