# Supplementary figures and images for: Exploring molecular markers and drug candidates for colorectal cancer through comprehensive bioinformatics analysis
Source: Aging (Albany NY). 2023 Jul 18;15(14):7038–55. doi: 10.18632/aging.204891 (PMC10415558; doi:10.18632/aging.204891)

SUPPLEMENTARY FIGURE

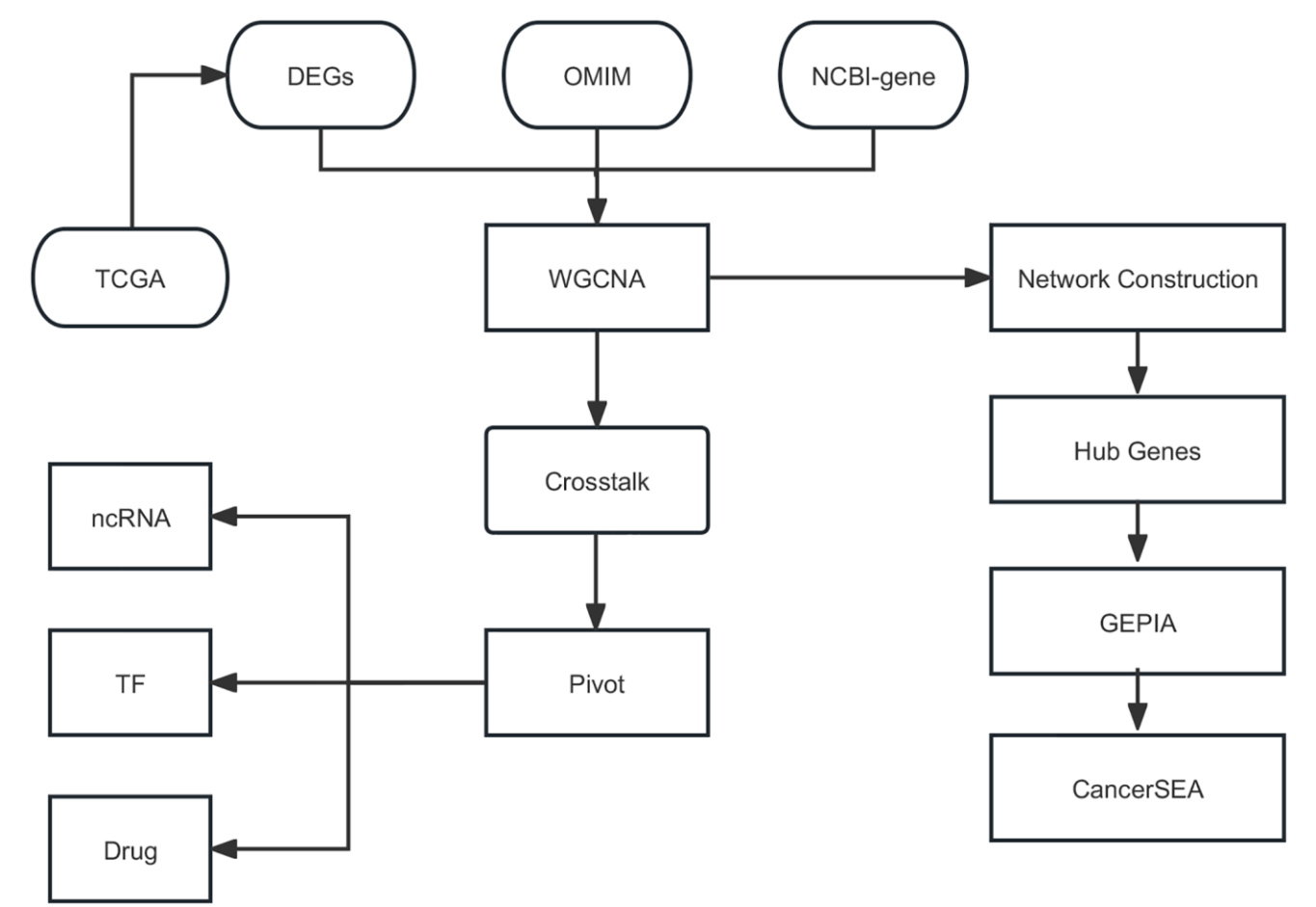

Supplementary Figure 1. Flowchart of this study.

Supplement: Supplementary Figure 1 [file aging-15-204891-s001.pdf]
